# Mapping the bacterial ecology on the phyllosphere of dry and post soaked grass hay for horses

**DOI:** 10.1371/journal.pone.0227151

**Published:** 2020-01-27

**Authors:** Meriel Moore-Colyer, Annette Longland, Patricia Harris, Leo Zeef, Susan Crosthwaite

**Affiliations:** 1 School of Equine Management and Science, Royal Agricultural University, Cirencester, Gloucestershire, United Kingdom; 2 Equine and Livestock Nutrition Services, Tregaron, Ceredigion, Wales; 3 Mars Horsecare United Kingdom LTD; Equine Studies Group, WALTHAM Centre for Pet Nutrition, Leicestershire, United Kingdom; 4 Faculty of Biology, Medicine and Health, University of Manchester, United Kingdom; 5 NIAB, EMR, East Malling, Kent, United Kingdom; University of Kentuky, UNITED STATES

## Abstract

Soaking hay fodder to reduce dust and soluble carbohydrate (WSC) contents prior to feeding is common practice among horse owners. Soaking can increase bacteria load in hay but no information exists on how this process alters the bacteria profile, which could pose a health risk or digestive challenge, to horses by introducing foreign bacteria into the gastrointestinal tract and so altering the normal profile. The current objectives were to map the bacterial profile of 3 different hays and determine how soaking alters this with the aim of improving best practice when feeding stabled horses. A Perennial Rye grass hay and two meadow s hays were soaked for 0, 1.5, 9 or 16 hours. Pre and post treatment, hays were analysed for WSC and total aerobic bacteria (CFU/g), and differences in bacteria family profiles were determined using ANOVA with significance set at P<0.05. Bacteria were identified *via* genomic DNA extraction and 16S library preparation (V3 and V4 variable region of 16S rRNA) according to the Illumina protocol. Differences in family operational taxonomic units (OTUs) between individual dry hays and different soaking times were identified *via* paired t-tests on the DESeq2 normalised data and false discovery rates accounted for using Padj (P<0.05). Mean % WSC losses and actual g/kg lost on DM basis (+/- SE) increased with soaking time being 18% = 30 (10.7), 38% = 72 (43.7), and 42% = 80 (38.8) for 1.5, 9 and 16 hours soak respectively. No relationship existed between WSC leaching and bacteria growth or profile. Grass type influenced bacterial profiles and their responses to soaking, but no differences were seen in richness or Shannon diversity indices. PCA analyses showed clustering of bacteria between meadow hays which differed from the perennial rye grass hay and this difference increased post soaking. Soaking hay pre-feeding causes inconsistent WSC leaching, bacteria growth and alterations in bacterial profiles which are unpredictable but may decrease the hygienic quality of the fodder.

## Introduction

Grass conserved as hay is used to feed a wide range of livestock and is still the preferred long forage for stabled equids in the UK [1], with 69.3% of owners feeding hay over the winter period [2]. However, in many temperate regions, it is difficult to make good quality hay both in terms of nutrient content and hygienic quality [3]. Alternatives to hay such as haylage and silage, while easier to conserve during a UK spring, are not always practical alternatives for horse owners. Small bales (15 -20kg) of commercially produced haylage are expensive relative to hay. The cheaper alternative of big bale haylage (200kg+), requires mechanical handling so is generally only suitable for larger yards with such equipment. Furthermore, once haylage is opened and exposed to the air, aerobic spoilage starts to occur with an increase in bacteria of 2.7 fold and mould of 6 fold noted by Leggatt and Moore-Colyer [4] after only 4 days post opening. The nutritional value and hygienic quality of hay depend on a plethora of factors such as grass species, fertilizer, edaphic and environmental conditions during growth and harvest, maturity at harvest and storage conditions. This makes hay not only highly variable in terms of nutritional content but can also present hidden challenges to the respiratory health of humans handling it and the animals consuming it [5,6,7].

Farmers Lung in humans and Equine Asthma in horses, are two well documented conditions arising from airborne respirable dust that is inherent in hay [7]. To reduce the airborne respirable dust 40.7% of owners soak hay before feeding [2]. The optimum soaking period for this purpose has been found to be 10 minutes [8], which maximises the reduction in airborne respirable dust (>95%) while minimising nutrient loss.

The second challenge when feeding hay is the variable water-soluble carbohydrate content (WSC) with some well conserved hays containing WSC contents in excess of 310 g/kg DM [9]. Such nutrient dense hays are not an issue for horses with high energy demands, but it makes these hays unsuitable to feed to animals pre-disposed to metabolic syndrome who should be fed forages of less than 100g/kg DM WSC [10].

Low WSC forage is often difficult to find, so owners of obese horses and those who have had laminitis are advised by their veterinary surgeons to reduce WSC content in the hay by soaking it. While the practice has previously been shown to be effective in reducing WSC, the degree of WSC loss is highly variable ranging from 9 to 54% [11,12] post soaking. Moreover soaking hay has some well-documented disadvantages such as leaching of Na, K and P [13] and production of post-soak liquor with a high biological oxygen demand [14] and recorded increases of 1.5 to 5 fold of bacteria, as measured by total viable count (TVC), in post soaked hay samples [15,16].

Bacteria can form large heterogeneous aggregates, reputed to constitute between 30 and 80% of the total bacteria on plant surfaces. Many of these aggregates also harbour fungi [17,18, 19], which may pose an additional threat to the hygienic quality of the fodder and the health of the animals consuming it. Identifying bacterial profiles of conserved forage will provide new insights as to which bacterial families commonly colonize cut fodder. Furthermore, a greater understanding of microbial profiles and interactions between bacteria the phyllosphere of herbage and pre-feeding soaking treatments may help horse owners decide on the best hay type and pre-feeding treatment to apply to the hay before feeding.

The objectives of this preliminary study were to map the resident bacterial profile (s) in 3 different types of hay when dry and to determine how that profile was altered by different soaking times. Additionally, the study also examined if any relationship existed between WSC leaching and the growth of bacteria in post-soaked hay. The aim of this research was to reveal information on bacteria in hay and to determine how pre-feeding treatments affect forage hygiene and thus add to best practice recommendations on feeding fodder to stabled horses.

## Materials and method

### Hay and sampling procedure

Three different hay types sourced from 2 different farms in Wiltshire were used in this experiment. 1 Perennial Rye Gress (PRG) and one meadow hay (MC) was sourced from Idstone Farm, Idstone, Ashbury, Wilts SN6 8LL; Lat N51:33:25, Long W1:37:51 and the second meadow hay (MS) came from Somerford House West Street, Great Somerford, Wiltshire, SN15 5EQ; Lat N51:32:37, Long W2:03:20. Three x 20kg (approximately) bales were collected of each hay type. As both are commercial farms and produce hay for sale to the public no permits were required for collection of the hay samples.

Hays were made in July 2013, were well conserved and had no visible signs of microbial spoilage. Bales had already been collected from the fields and were stored in Dutch-barns. Three bales of each type of hay were collected from different parts of the stacks, avoiding the edges to avoid any effects of weathering during storage. As all farmers and horse owners appreciate, individual hay bales from one field can vary quite considerably, due to edge effects, soil differences and species composition differences (the latter especially in meadow hay). Therefore, the three individual bales were treated as separate samples (nested within) the hay type, and were representative of variation that horse owners face on a daily basis. Results from the 3 bales for each hay type were not, therefore, averaged as they could not be treated as exact replicas of each other.

Post-purchase, all bales were labelled and stored off the ground on pallets in a wooden building at the Royal Agricultural University. Each individual hay bale was subjected to the following procedure:

Individual bales were opened and spread out on a new, clean plastic sheet in an enclosed glass house. Using gloved hands and wearing a protective mask, the hay was teased apart and thoroughly mixed before subsamples of 2kg each were placed into 4 individual small-holed hay nets. Hay nets were then put into purpose made, pre-labelled, new polyester hay bags (Haygain Ltd, Hungerford, Berkshire UK), and stored until treated. The remaining hay from each bale (approximately 12 kg) was stored in a labelled clean polyester hay bag for later use for bacterial DNA extraction.

### Treatments

The 4 x 2 kg hay nets from each bale were treated as follows: Hay net 1 was left dry (D) where no additional treatment was applied to the hay; Hay net 2was Soaked (1.5) by total immersion in 30 litres of clean tap water at 16°C for 1.5 hours, then hung up to drain for 10 minutes; Hay net 3 was Soaked (9) by total immersion in 30 litres of clean tap water at 16°C for 9 hours, then hung up to drain for 10 minutes; and Hay net 4 was Soaked (16) by total immersion in 30 litres of clean tap water at 16°C for 16 hours, then hung up to drain for 10 minutes; The procedure was repeated for bales 2 and 3 for each of the 3 hay types.

Post-treatment, the hay from each hay net was mixed by gloved-hand and two sub-samples were taken. Sub-sample 1 (approximately 10 g) was placed into a sterile plastic bag and placed in a laminar-flow cabinet (Bassaire, Duncan Rd, Swanwick, Southampton), for bacterial culturing. Sub-sample 2 (approximately 800 g), was weighed onto a new pre-weighed foil tray and placed in a forced draught oven. Samples were dried at 60°C to minimise the formation of Maillard products which can occur at temperatures of > 70°C, and dried until a constant weight was reached for dry matter (DM) determination. Sub- sample 2 having been dried was then milled through 0.75 mm mesh using a 1093 Cyclotec Sample Mill (Foss Sweden) and 50 g of the dried, milled sample was retained and stored in sterile plastic tubes (VWR, UK) for subsequent WSC analyses, the remaining dried sample was placed in sterile plastic bags and put in the freezer.

### Bacterial culturing and enumeration

Immediately post-treatment, sub-sample 1 was roughly chopped into 2cm lengths with scissors, (previously wiped with ethanol, and allowed to dry) and thoroughly mixed. A one gram sub-sample was then weighed into a sterile plastic bag (Seward BA6040) to which 79 ml of sterile peptone saline solution (MRD) was added. The peptone solution was prepared using 1g/l enzymatic digest of casein and 8.5g/l sodium chloride (VWR https://sg.vwr.com/cms/about_vwr, chemicals, catalogue number 84617) placed into flasks and sterilised in autoclave at 121°C for 15 minutes; (The bag was then placed into a Lab Blender 80 model (Steward Laboratory, Blackfriars Rd, London). The mixture was then ‘blended’ for 2 minutes in order to wash bacteria from the hay into the solution as for 3M petrifilms (3M Microbiology, 2013). One millilitre of the blended solution was placed into a sterile screw-cap tube (VWR, UK) containing 9 ml MRD. Serial dilutions were prepared to 10^−6^. A 1 ml sample was then taken from 10^−2^, 10^−4^ and 10^−6^ dilutions and separately placed onto pre-labelled 3M Aerobic total viable count (TVC) 20 cm^2^ petrifilm, (3M Microbiology, Carl-Schurz-Straβe 1, Germany). Petrifilms are a sample ready culture medium, containing nutrients, a cold water-soluble gelling agent and a tetrazolium indicator. Three petrifilms were prepared for each sample and incubated for 3 days at 32°C.

Colony numbers were enumerated using an illuminated magnifier. All vital stained colonies were counted. When colony numbers were particularly dense and small and >100 per film, three 1 cm squares away from the edges of the film were counted. The average was determined, and scaled up 20-fold as an estimation of the count per film as per the manufacturer’s instructions.

### Water soluble carbohydrate analyses

The WSC content of each hay pre- and post-soaking (3 replicate determinations per sample) were determined by the Phenol-Sulphuric Acid method [20]. To summarise, 3 x 100 mg samples of each dried and ground hay were separately placed in 3 x 25 ml volumetric flasks. De-ionised water at room temperature was added to each of the flasks and made up to the mark, and flask contents were mixed by inversion and again at 20-minute intervals over the course of an hour. Flask contents were then individually filtered through a Whatman no 1 filter paper into separate, 100ml volumetric flasks and the filtrates made up to the mark with deionised water. One millilitre of each filtrate was placed in a boiling tube to which 1 ml of 5% (W/V) aqueous phenol was added, followed by the swift addition of 5ml of concentrated sulphuric acid. Tube contents were then mixed on a vortex mixer. Thirty minutes later, the absorbance was read against a reagent blank at 490 nm. A standard curve was prepared using glucose (10–100 μg ml^-1^).

### Preparation of hay for DNA extraction and sequencing

DNA extraction was carried out at the Faculty of Biology, Medicine and Health, University of Manchester. As samples had to be booked in to be sequenced and the distance between sites was a 3-hour drive, there was a delay between soaking treatment and sequencing. Frozen samples can show deterioration of bacterial DNA, thus it was decided to take dry hay samples to the lab and replicate the soaking treatments on a bench-top scale, before extraction of DNA. So the remaining 12 kg of stored dry hay from each bale was sub-sampled, taking approximately 100 g from each replicate bale, thus 300g of each hay type was used. Each 100g replicate sample underwent the following procedure. A 0.5g sub-sample was placed in a 50 ml glass tube. Seven and a half ml of tap water at 16 ^o^C was added to each tube, covered with foil and placed in an incubator at 16°C. After soaking, for 0, 1.5, 9 or 16 hours, samples were placed on Whatman filter papers for 10 minutes and then chopped into approximately 0.5 cm lengths for DNA extraction.

Genomic DNA was extracted using the MoBio PowerMaxSoil TM kit (MoBio, Carlsbad, CA, USA) according to the manufacturer’s protocol. 16S library preparation was carried out according to Illumina’s protocol. Briefly, genomic DNA was amplified with forward primer 5’-CGTCGGCAGCGTCAGATGTGTATAAGAGACAGCCTACGGGNGGCWGCAG-3’ and reverse primer 5’ -GTCTCGTGGGCTCGGAGATGTGTATAAGAGACAGGACTACHVGGGTATCTAATCC-3’ targeting the V3 and V4 variable region of the 16S rRNA gene [21,22]. Twenty five microlitre PCR reactions contained 12.5 μl 5 KAPA HiFi HotStart Ready Mix Master Mix, 5 μM final concentration of forward and reverse primers and 21 ng gDNA. Amplification program: 95°C 3 mins, 25 cycles of 95°C 30 s, 55°C 30 s, 72°C 30 s, final extension 72°C 5 min. A subsequent limited-cycle amplification step was performed to add multiplexing indices and Illumina sequencing adapters. Ampure XP beads were used in the PCR clean-up after 1^st^ and 2^nd^ stage PCR. The libraries were then normalised, pooled and sequenced on the MiSeq platform. Reads for this study were submitted under ENA Study Accession No.: ERP118485. The quality of raw sequence data was first checked using FastQC. Next, Trimmomatic was used to filter out poor reads, sequences were truncated to 180 bp and paired ends joined using SeqPrep [23]. Sequences were uploaded to QIME [24] where they were clustered into operational taxonomic units with a 97% similarity cut off using as a reference the pre-clustered versions of the Greengenes database [25]. OTUs with only a single representative read were removed.

Principal Component Analyses (PCA) and differential counts between conditions were performed. PCA 1 = strongest pattern of variance and PCA 2 the second strongest pattern of variance. Differences between all 3 hay types and 4 wetting treatments were performed on DESeq2 normalised data using ANOVA (Genstat 18) and LSD = t _(error df)_ x s.e.d. and significance taken at P<0.05. In order to determine if any of the soaking times significantly affected the bacteria content within individual hay types, paired t-tests between D and 1.5, D and 9 and D and 16 hours were calculated for each hay type. In order to take account of false discovery rates that can occur in such data sets with multiple parallel measurements, significance determined using Padj value of <0.05 was taken as the cut-off point for differences.

### Bacterial diversity, richness and similarity between hay types and treatments

Shannon diversity indices, and richness tests were calculated on the *ca* 250 bacterial family OTUs identified for all hays and treatments. Difference in diversity and richness across hay types and treatments were compared using ANOVA and significance set at P< 0.05.

Jaccard similarity index (Ji) using the formula: Ji = X˄Y/X˅Y, which is a simple % similarity value calculated between two samples was used to determine the level of commonality between the PRG and MS, PRG and MC and MS and MC hay types and within individual hay types between dry and 1.5, 9 and 16 hours soaking treatments.

In order to determine if soaking for different times has any effect on bacterial profiles in the three different hays matched pairs tests were carried out at the family level within hay type. Therefore t-tests were done between MCD vs MC1.5, MCD vs MC9 and MCD vs MC16; MSD vs MS1.5, MSD vs MS9 and MSD vs MS16; PRGD vs PRG 1.5, PRGD vs PRG9 and PRGD vs PRG16.

### Data analyses and sample size

Based on previous data from hay soaking experiments a power calculation showed that 3 replicates would be sufficient to detect a commonly recorded 35g difference in WSC content post-soaking. WSC and bacteria (TVC) data were tested for normality using Q-Q plots in Genstat 18. Differences in WSC and colony forming units of bacteria (CFU/g) contents between the three different hays when dry were determined using analysis of variance (ANOVA), with hay types (3), replicate bales (3) as fixed factors as per the method of Moore-Colyer et al [26]. Differences between means were calculated using least significant difference (LSD) test where LSD = t _(error df)_ x s.e.d. and significance taken at P<0.05. Differences between the 4 different wetting treatments within hay type for both WSC and CFU were also determined using ANOVA on log 10 transformed data using Genstat 18 as described by the procedure for skewed data [27]^.^ Differences between treatment means were determined using least significant difference (LSD) test where LSD = t (error df) x s.e.d and significance taken at P<0.05. Results for WSC contents were expressed as g/kg on a DM basis, while those TVC were expressed as geometric mean colony forming units (CFU)/g on an as fed basis, as this value approximates closely to the median [27] which is widely accepted to be the most accurate expression of the distribution of the CFU in the samples. Linear regression analysis was performed in excel to test if any relationship existed between WSC content and log 10 CFU/g of bacteria.

## Results

### Dry matter, water soluble carbohydrate, bacteria content and profile in dry hay

In this preliminary study, three hays that were specifically made for horses in the Southwest of England were chosen to represent commonly purchased horse hay. A complete growing and harvest history was not available, and no nutritional or hygienic analyses was done pre-purchase; the only quality assessment was done by sight and smell.

On visual assessment the dry MCD and MSD hays contained similar varieties of grass species inclusive of perennial ryegrass, rough stalked and annual meadow grasses, Timothy, Yorkshire fog, Cocksfoot, and small amounts of Crested Dogs Tail, the proportions of these were not accurately determined. The hays were well conserved, were slightly green in colour and smelled sweet and were all above 85% DM as shown in Table 1. None of the hays had been rained on post cutting and post-harvest all bales were stored in open-sided Dutch barns.

**Table 1:** Differences in dry matter (DM), and water soluble carbohydrate (WSC) content (g/kg DM) and bacteria content (log 10 CFU/g) of 2 meadow (MCD and MSD) and 1 perennial rye grass (PRGD) hays when dry.

	MCD	MSD	PRGD	sed	Sig
**Dry matter (DM) (g/kg)**	93.2^a^	96.6^b^	92.6^a^	0.32	0.001
**WSC (g/kg DM)**	242.6^b^	124.6^a^	204.3^b^	17.76	0.006
**Bacteria (log 10 CFU/g)**	8.06	6.99	7.32	0.765	0.457

ab Values in the same row not sharing common superscripts differ significantly (P<0.05)

The differences in WSC contents as determined by 1-way ANOVA of the three dry hays i.e., before treatment detailed in Table 1 show that MSD hay was lower (P<0.05) in WSC content than the other two hays being 118 and 80 g WSC/kg DM lower than MCD and PRGD respectively. There was no difference in the abundance of bacteria between the hays. The geometric means within hay type CFU/ g revealed high contents of bacteria for all the hays (S1 Table).

Jaccard similarity index on the dry hays before treatment revealed that there was an 81% similarity in bacteria families between PRGD and MCD, an 87% similarity between PRGD and MSD, and an 86% similarity between MSD and MCD. Similarities between soaking treatments within hay type was lowest (81%) for PRG between PRGD and PRG1.5 and highest (93%) between MCD and MC 9 (S2 Table)

**Fig 1:**
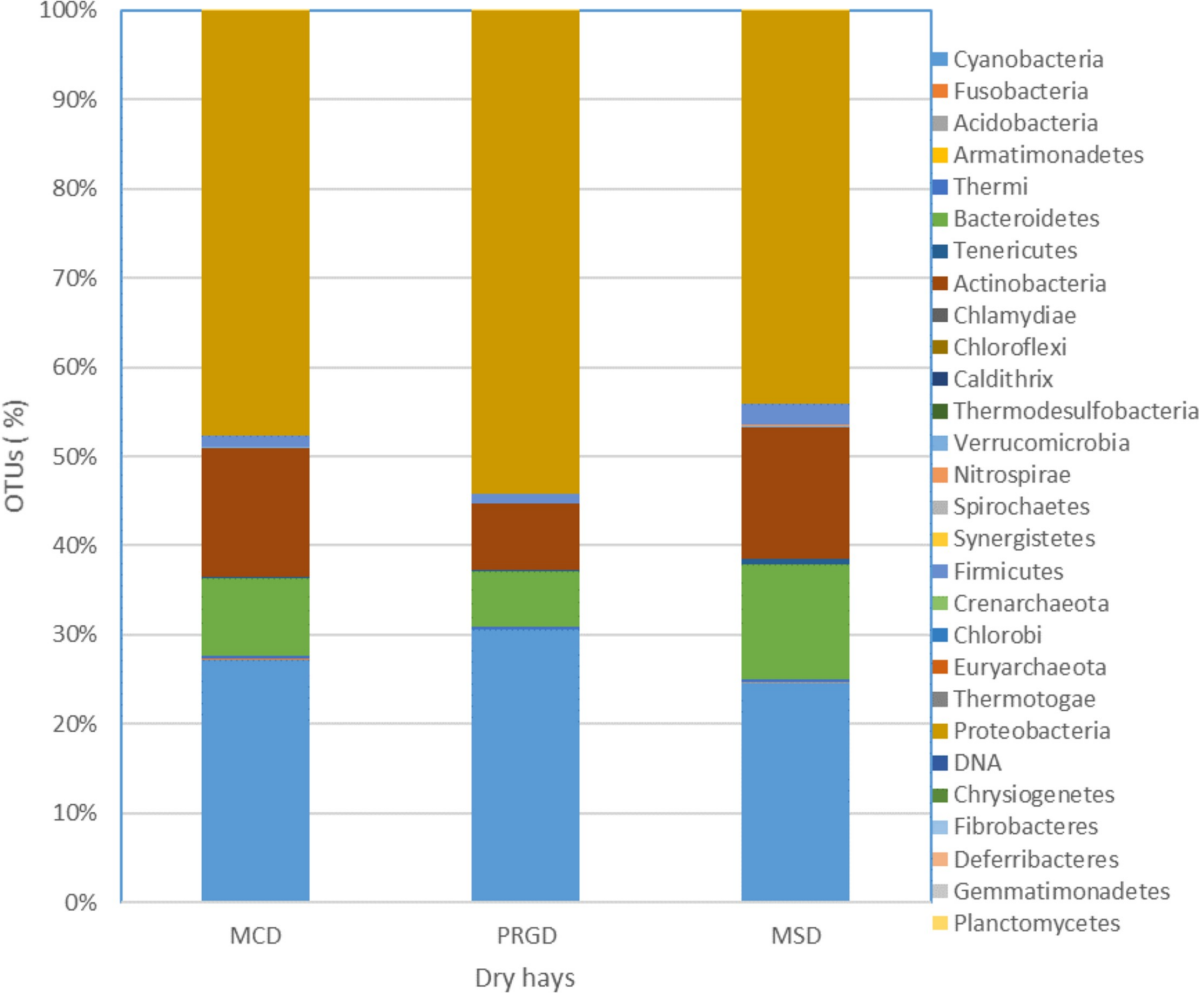
Percentage of operational taxonomic units (OTUs) of bacteria phyla present in 2 types of dry meadow hay (MCD and MSD) and dry perennial rye grass hay (PRGD).

Across all three hays a total of 28 phyla, 265 families and 712 genera were identified (S3 Table). All 28 phyla were present in MCD and 26 in PRGD and MSD. The profile of bacteria phyla in all three dry hays are shown in Fig 1 representing percentages of operational taxonomic units (OTUs). The 4 phyla that represented >96% of the bacteria present were Proteobacteria (44–54), Cyanobacteria (24–30), Actinobacteria (074–14) and Bacteroidetes (062–13). Although the proportional profiles of Actinobacteria and Bacteriodetes were double in the MCD and MSD hays compared with the PRGD hay, none of these differences were significant (S4 Table).

**Fig 2:**
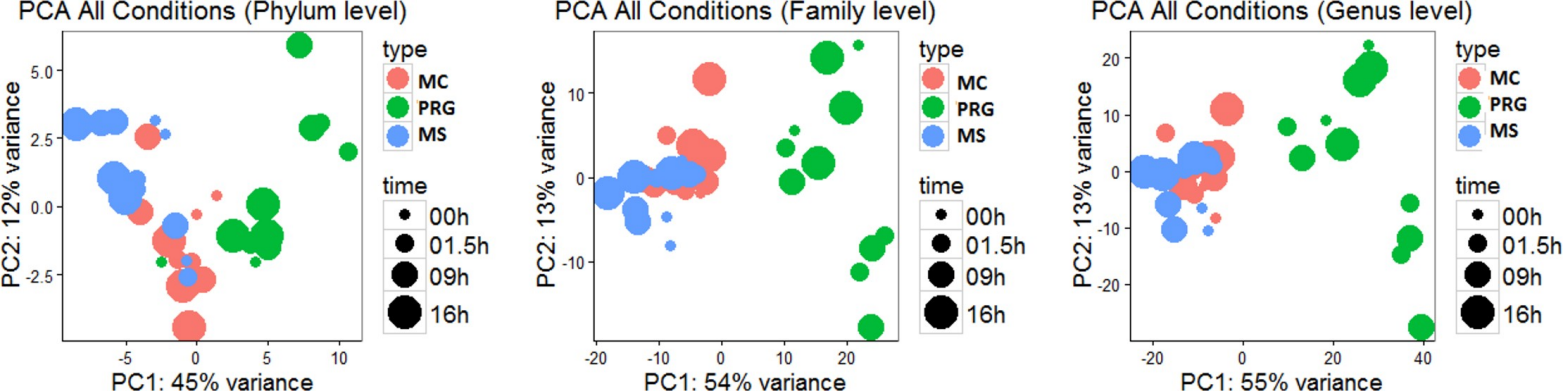
Principal component analyses of bacteria phyla, family, and genus for the three hays MC (C), PRG (P) and MS (S) when dry and after soaking for 1.5, 9 and 16 hours.

The other phyla identified comprised less than 4% of the proportion of OTUs in each hay. Fig 2 shows the principal component analyses across the three hays and the 4 different wetting treatments of dry (D), 1.5 hr soak (1.5) 9-hour soak (9) and 16-hour soak (16). There was a degree of similarity between the bacteria families in the dry and post-soaked samples for the two meadow hays (MC and MS) but families present in pre and post-soaked PRG were clearly different.

### The effect of soaking time on the dry matter, water soluble carbohydrate and microbial numbers (CFU/g) in hay

Post-soaking, the forages absorbed between 50 and 62% additional moisture with no pattern emerging according to soaking time. The effect of soaking time on WSC content across all 3 hay types is shown in Table 2. The average WSC loss across all hay types for the 1.5 hours soaking period was 34 (+/-46.1) g/kg DM. Soaking for 9 or 16 hours caused a 72 (+/-23.8) g/kg DM, an average loss of 38% of the WSC present in the dry hays. Variation in WSC loss was high ranging from 6 to 30% for 1.5 hours, 17–54% for 9 hours and 31–63% for 16 hours soaking.

**Table 2:** Mean Water Soluble Carbohydrate (WSC) content (±SD) across all 3 hays when dry (D) and after soaking in water for, 1.5, 9 and 16 hours at 16°C.

Soaking time	D	1.5	9	16	sed	Sig
**WSC content (g/kg DM)**	190.5^c^	156.8^b^	118.4^a^	110.6^a^	11.40	0.001
**SD**	54.6	46.1	23.8	32.8		
**Range % loss**		6–31	17–54	31–63		

^abc^ Values in the same row not sharing common superscripts differ significantly (P<0.05)

When considering each hay individually, (Tables 3–5) the impact of soaking on the WSC content in MS hay, the duration of soaking had no impact on WSC loss. However, differences were noted when MC (Table 4) was soaked with a 17% loss post-soaking for 1.5 hours and a further 39% loss when soaking was extended to 9 and 16 hours. PRG (Table 5) showed a similar pattern to MC with 19% losses for the 1.5 hour soak increasing to 50% and 60% respectively for the 9 and 16 hour soaks.

**Table 3:** The effect of 4 different soaking times dry (D), 1.5, 9 and 16 hours (±SD) on the dry matter (DM), bacteria content log CFU/g (±SD) and as geometric mean (CFU/g) and water soluble carbohydrate (WSC) in Meadow hay (MS).

parameter	MSD	MS1.5	MS9	MS16	s.e.d	Sig
**DM**	96.6 (±1.4)	49.1 (±6)	44.4 (±3.4)	45.7 (±1.4)		
**% H**_**2**_**0 content**	3	51	56	54		
**Log CFU/g**	6.99 (±0.78)	7.36 (±0.15)	7.47 (±0.05)	7.47 ±0.18)	0.284	0.364
**Geo mean CFU/g**	2.4 x10^7^	2.3 x10^7^	2.9x10^7^	3.1x10^7^		
**WSC**	125(±23.6)	104(±6.3)	103(±6.3)	97(11.4)	12.27	0.225
**% WSC loss**		17	18	23		

**Table 4:** The effect of 4 different soaking times dry (D), 1.5, 9 and 16 hours (±SD) on the dry matter (DM), bacteria content log CFU/g (±SD) and as geometric mean (CFU/g) and water soluble carbohydrate (WSC) in Meadow hay (MC).

parameter	MCD	MC1.5	MC9	MC16	sed	Sig
**DM**	93.2(±1.2)	48.7(±2.9)	46.8(±4.4)	43.0(±3.4)		
**% H**_**2**_**0**	7	51	50	54		
**Log CFU/g**	8.06(±1.15)	7.49(±0.09)	7.62(±0.08)	7.53(±0.23)	0.510	0.674
**Geo mean CFU/g**	76 x10^7^	3.1x10^7^	4.2x10^7^	3.6x10^7^		
**WSC**	242.6^a^(±6.8)	199.6^b^(±124)	148.5^c^(±13.6)	151.8^c^(±13.8)	15.37	0.003
**% WSC loss**		17	39	38		

**Table 5:** The effect of 4 different soaking times dry (D), 1.5, 9 and 16 hours (±SD) on the dry matter (DM), bacteria content log CFU/g (±SD) and as geometric mean (CFU/g) and water soluble carbohydrate (WSC) in Perennial Rye Grass hay (PRG).

parameter	PRGD0	PRG1.5	PRG9	PRG16	sed	Sig
**DM**	92.6(±1.6)	36.8(±5.9)	35.0(±3.5)	42.8(±12.6)		
**% H**_**2**_**0**	7	60	62	54		
**Log CFU/g**	7.30(±0.57)	7.42(±0.00)	8.15(±1.01)	7.55(±0.03)	0.416	0.289
**Geo mean CFU/g**	3.7x10^7^	2.6x10^7^	71.5x10^7^	3.5x10^7^		
**WSC**	204.3^a^(±21.9)	166.8^b^(±20.3)	103.7^c^(±3.3)	83.0^c^(±4.5)	12.36	0.001
**% WSC loss**		19	50	60		

^abc^ Values in the same row not sharing common superscripts differ (P<0.05)

**Fig 3:**
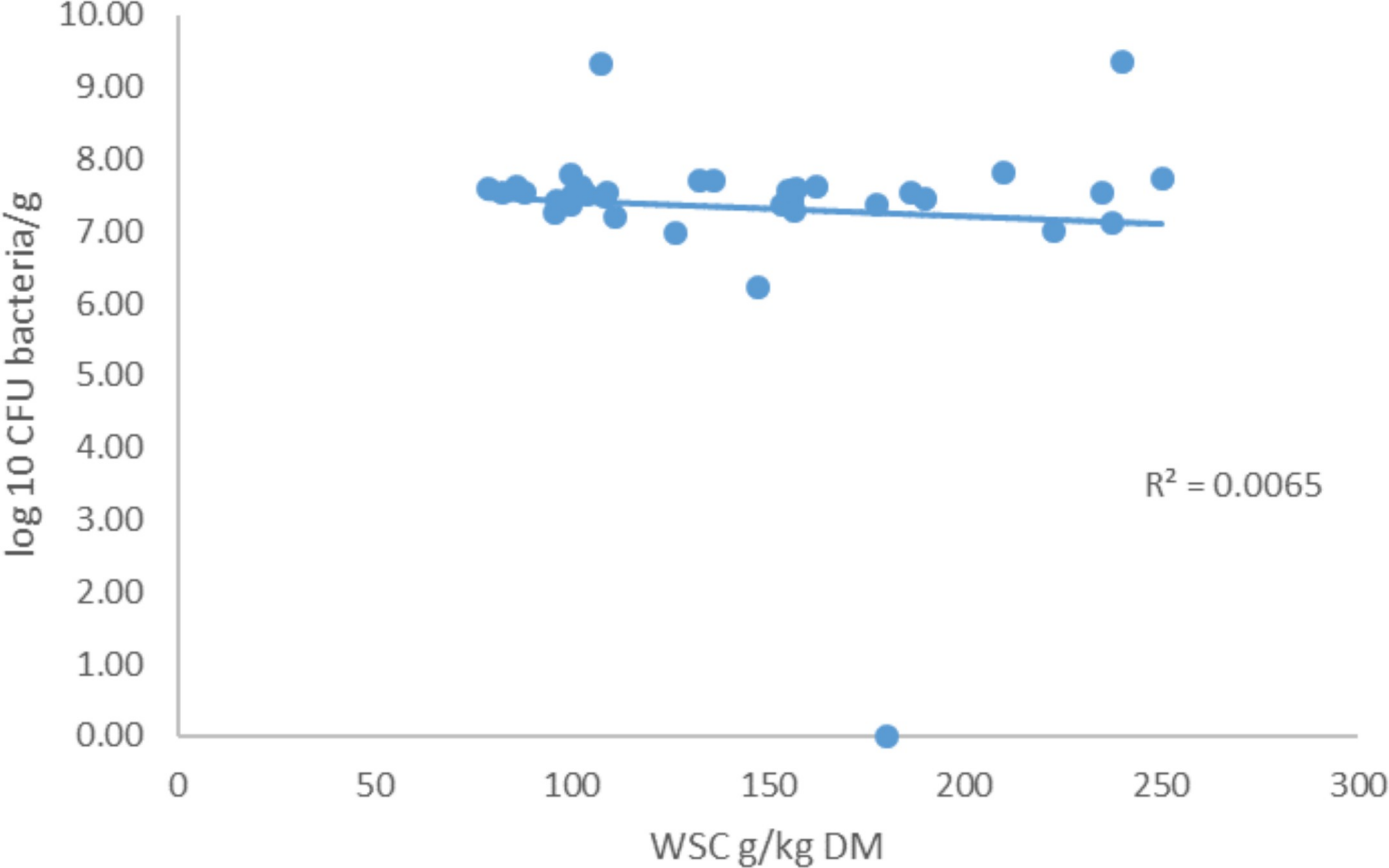
Relationship between WSC loss (g/kg DM) and bacteria CFU/g across all hays and all treatments.

Fig 3 shows the relationship between WSC loss and log 10 CFU g/kg DM of bacteria. The r^2^ value was 0.0065 showing that bacteria content was not influenced by WSC leaching in these three hay types.

### Effect of soaking for 1.5, 9 and 16 hrs on bacteria phyla and family profiles across all hay types

The sequencing across the 36 samples (3 hays x 3 replicates x 4 treatments) gave 24720 OTUs identified and produced 31786294 total counts. The mean counts per sample were 882952.6 (± 389587) and ranged from 281311 to 1708354. As shown in Table 6, across all three hay types, soaking decreased (P<0.05) the OTUs for the phyla *Cyanobacteria* with OTUs highest in D > 1.5 > 9 = 16. In contrast, *Deferribacteres* increased (P<0.05) with soaking time with 16 > 9 = 1.5 > D. All other bacteria phyla were not influenced by soaking time. Across all three hays, neither the mean richness nor the Shannon Diversity Index (Table 7) was influenced by soaking time.

**Table 6:** The effect of 4 different wetting treatments on the profile of bacteria phyla denoted by OTUs (and % when greater than 1) across all 3 types of horse hay: perennial rye grass hay (PRG), and two types of meadow hay (MC and MS).

Phyla	Dry	Soaked 1.5 hours	Soaked 9 hours	Soaked 16 hrs	sed	Sig
**Acidobacteria**	93	132	482	386	253.6	0.407
**Actinobacteria**	136294 (12.2)	120456 (11.4)	141336 (13.1)	151622 (9.2)	39982.7	0.885
**Armatimonadetes**	99	78	161	174	82.5	0.613
**Bacteroidetes**	108292 (9.3)	92265 (8.6)	129958 (11.8)	189237 (11.4)	43917.5	0.236
**Caldithrix**	3.3	6	9	6.7	2.85	0.348
**Chlamydiae**	66	47	109	69	35.8	0.429
**Chlorobi**	4.67	5.00	4.67	4.00	1.089	0.827
**Chloroflexi**	247	295	189	294	71.1	0.456
**Chrysiogenetes**	2.67	2.33	2.33	3.67	1.298	0.713
**Deferribacteres**	0	1.00	0.33	0.33	0.360	0.138
**Cyanobacteria**	315575^c^ (27.5)	287465^b^ (29.2)	176633^a^ (17.0)	140057^a^ (7.2)	51456.2	0.038
**Deferribacteres**	7.9^a^	10.9^a^	10.3^a^	23.8^b^	3.57	0.016
**Euryarchaeota**	1.41	0.84	0.73	0.79	0.424	0.416
**Firmicutes**	19018 (1.6)	18799 (1.8)	30781 (2.8)	15910 (1.0)	5929.8	0.159
**Fusobacteria**	183	586	4140	132	2737.0	0.457
**Gemmatimonadetes**	0.57	1.60	2.00	2.72	1.711	0.664
**Nitrospirae**	258	290	194	240	71.7	0.628
**Planctomycetes**	166.8	169.8	154.9	155.1	15.29	0.685
**Proteobacteria**	559956 (48.6)^a^	499615 (48.0)^a^	577340 (54.0)^a^	1273042 (70.0)^b^	252292.3 5.05	0.064 0.014
**Spirochaetes**	30.4	28.2	17.3	18.1	6.53	0.197
**Synergistetes**	9.48	6.31	9.21	6.62	1.271	0.091
**Tenericutes**	4079	4122	4605	3916	506.5	0.593
**Thermi**	3950	3547	2888	4076	1527.5	0.862
**Thermodesulfobacteria**	48.1	111.6	79.0	40.8	27.25	0.129
**Thermotogae**	14.7	16.7	23.6	17.8	5.59	0.482
**Verrucomicrobia**	1175	863	1629	1253	313.3	0.213
**Total number of OTU sequences**	3,448,756	3,086,811	3,212,293	5,342,137		

^abc^ Values in the same row not sharing common superscripts differ significantly (P<0.05)

**Table 7:** Mean richness of bacteria families and Shannon Diversity Index (H) for the four different soaking times of dry (D), 1.5, 9 and 16 hours across all three hay types.

Soaking time	D	1.5	9	16	sed	sig
Richness	225	222	232	250	7.08	0.493
H index	2.47	2.47	2.93	2.37	0.194	0.093

### Effect of soaking for 1.5, 9 and 16 hrs on bacteria families within hay types

**Fig 4:**
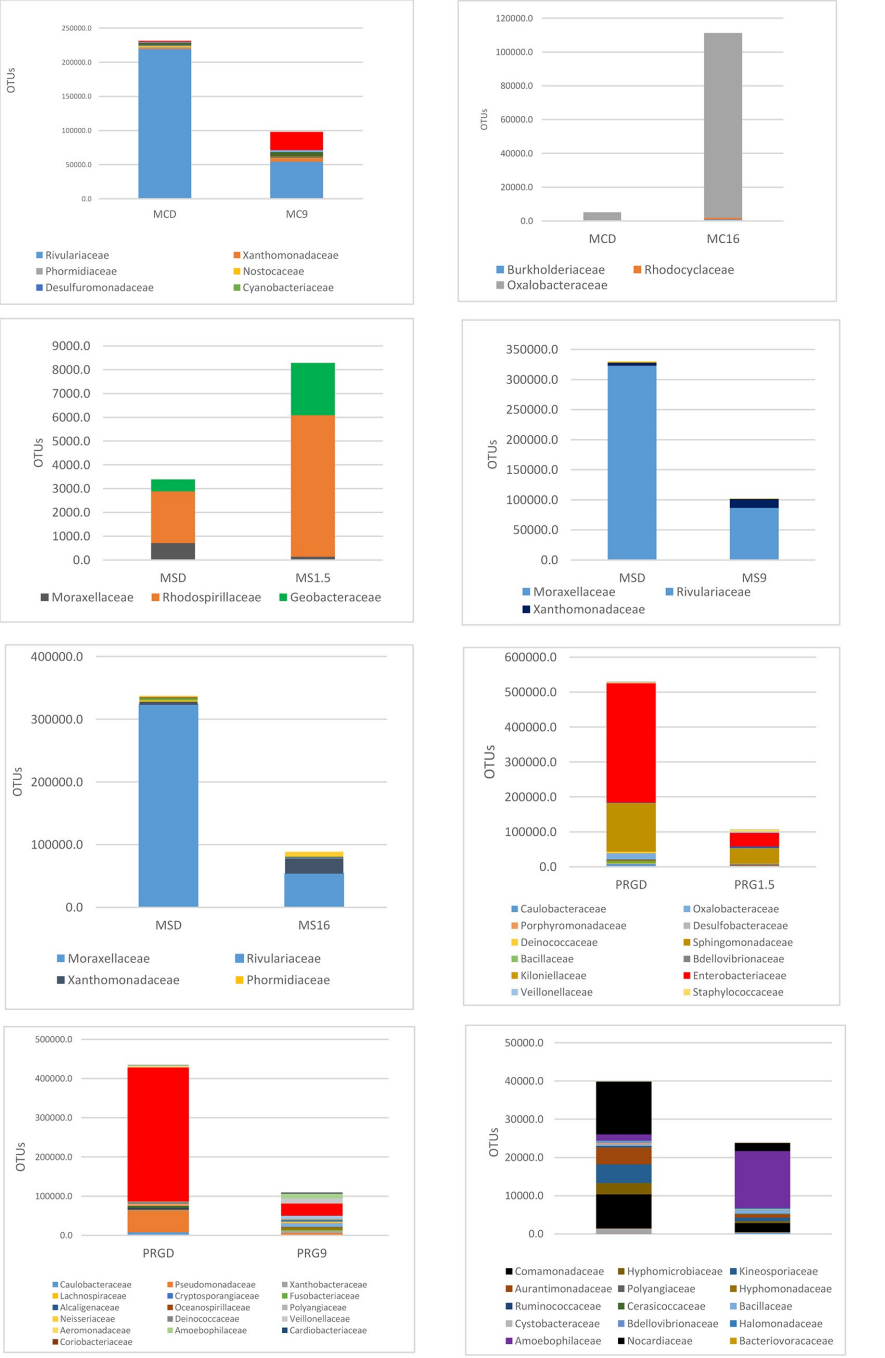
Differences (P<0.05) within hay type between dry and the three soaking treatments of 1.5, 9 and 16 hours on bacteria family profiles as measured by operational taxonomic units (OTUs).

Fig 4 highlights the significant alterations post soaking, in the bacteria families that formed a major component of the bacteria present in the dry hays (S5 Table).

Looking at the abundance of bacteria across the different hay types, the influence of soaking time did not produce any clear pattern in bacteria reduction or increase. No differences at all were seen between MCD and MC1.5. Soaking the MC9 caused an increase in the Enterobacteriaceae family but decreased the Rivulariaceae while the longer soaking time of 16 hours produced an increase in Oxalobacteraceae. With the MSD vs MS1.5 both Rhodospirillaceae and Geobacteraceae were increased, no difference was seen between MSD and MS9 and an increase in Xanthomonadaceae but a decrease noted for Rivulariaceae for the MSD vs MS16. PRG D vs PRG 1.5 showed a decrease in Enterobacteriaceae, no difference when soaking from 9 hours and an increase in Amoebophilaceae when soaking for 16 hours.

**Fig 5:**
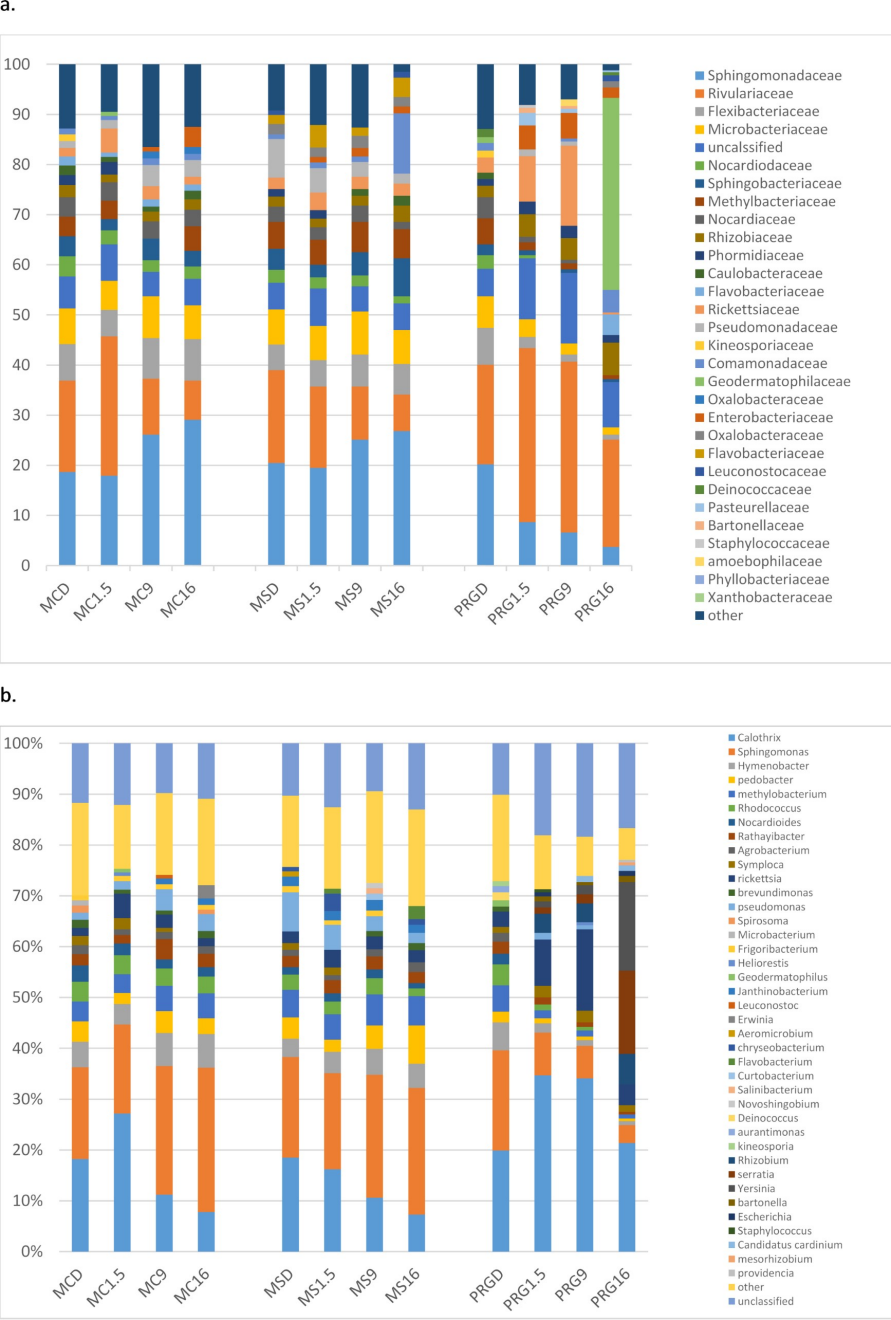
The profile of bacteria in the three hays when dry, soaked for 1.5 hours, 9 hours and 16 hours. a. major bacteria families, b. major bacteria genera.

Fig 5 shows the bacteria profile within each hay type in major bacteria families (Fig 5A) and genera (Fig 5B) when dry and post 1.5, 9 and 16 hours soaking. All three hays contained similar bacteria, but a different proportions. While bacteria species appeared to alter upon soaking variation between replicate samples resulted in no significant differences in the genera being present within the hays post- soaking.

## Discussion

### Dry hay, water soluble carbohydrate content and microbial colony forming units (CFU) / g

All hays were well conserved and had DM of > 85%, which is recommended to ensure good crop conservation [28]. The range of WSC in the three hays of 125 to 242 g/kg DM is typical of UK hay and agrees with those quoted by previous researchers [12, 29]. MC and PRG hays contained 100 to 142g/kg DM more WSC than is currently recommended for forages intended to be fed to obese equids or those with a pre-disposition to laminitis. The forages used in this study were therefore representative of those that horse owners commonly soak in water, in order to reduce the WSC content before feeding. Although there were no visible signs of aerobic spoilage in any of the hays, the bacteria CFU / g were notably higher than the 3.0 x10^7^ CFU/g recommended by Bucher and Thalman [30] and in previously published findings for a range of single and mixed species hays [26, 31]. It has been noted by Behrendt *et al*. [32] Muller *et al*.[33] that late harvesting can increase the microbial load in forages and all 3 hays used in this study were harvested in late July which may partially explain the high bacteria levels.

### Profile of bacteria in dry hays using 16S rRNA sequencing

Lindow and Brandl [34] noted that bacteria are by far the most numerous colonists of plant leaves, often being found in numbers up to 10^8^ cells/g of leaf [35, 36]. While yeasts are active and effective colonizers, filamentous fungi in the form of spores are more transient occupants than bacteria. Commonly the study of bacteria on the leaves of plants has been driven by their deleterious effect on plant productivity and has been largely restricted to aerobic culturable gram negative bacteria, particularly *Pseudomonas spp*. *(syringae)* and *Enterobacteriaceae (Erwinia*, *Pantoea*), which are two of the most ubiquitous bacteria colonizers of the phyllosphere [34]. Although no studies detail the bacteria profile on grass hay, a study of bacteria on the phyllosphere of grasses growing in extensive pastures found the most prominent 5 genera (phylum in brackets) were *Pseudomonas* (Proteobacteria), *Stenotrophomonas* (Proteobacteria), *Pantoea* (Proteobacteria), *Clavibacter* (Actinobacteria) *and Curtobacterium* (Actinobacteria) [31]. The bacteria genera identified in the grass hays in this study also contained *Pseudomonas* and to a minor extent *Curtobacterium*. The other bacteria that might be expected to be present on grass is the genera *lactobacillus* (Firmicutes). These bacteria play a major role in fermenting grass into silage, however, although 19 genera (5 identified down to species level) were sequenced they were in low abundance and did not alter with soaking treatment. *Yersinia* was also noted in the hay samples in this study and may have come from rodent contamination during storage; it was not noted to be present on the growing grasses in the study by Behrendt *et al*., [32]. These differences are not surprising as epiphytic bacteria populations can differ in size and profile between plant species and within plants of the same species. Furthermore, changes in bacteria populations on the growing plant can be rapid and are influenced by a wide range of factors such as physiological age, macro and micro environmental conditions, and on-leaf microbial interactions [37, 38]. Such factors could readily explain the differences within species, between conserved and growing grasses and between forage types as noted here.

The small non-significant variations in phyla profile, richness, and diversity, particularly between the PRG and the two meadow hays may hint at real differences which were not detected due to the small sample size used in this study. Physical and nutritional conditions on the phyllosphere can account for considerable variations in plant microbial carrying capacity. PRG has a shiny under leaf and bacteria establishment and maintenance can be affected by glossy mutants with the fewest crystalline waxes. Such leaves prove a less effective host for epiphytic bacteria than those with less shiny cuticles [39] and the tendency for a greater diversity in the meadow hays compared with the PRG hay noted here supports this.

### The effect of soaking time on the dry matter (DM) and water soluble carbohydrate (WSC) contents of the hays and their bacteria numbers and profiles in hays

The absorption of water post soaking of between 50 and 62% noted here were slightly lower than the 73% recorded by Moore-Colyer *et al*., [26] for a range of Meadow, Timothy and Italian Rye grass hays also soaked for 9 hours, which may have affected WSC leaching.

Extended soaking periods of between 9 and 12 hours have been recommended by Longland, *et al*., [12] and Muller *et al*., [40] as a method by which to reduce the WSC content of fodder intended to be fed to horses with insulin resistance, metabolic syndrome, laminitis or obesity. Muller [40] recorded an average WSC loss of 43% after a 12-hour soak; and the losses recorded in the present study of 18, 38 and 42% after 1.5, 9 and 16 hours soaking are in broad agreement with these values. However, such losses cannot be predicted nor relied upon as variability of loss across the hays was high 6–63% and echo the caution expressed by Longland *et al*., [12] who recorded variations in WSC leaching from a variety of hays of 9 to 54% after a 16-hour soak. The present study reported no additional benefit in terms of WSC leaching when hay was soaked for longer than 9 hours. Furthermore no pattern was evident in the present study between initial WSC content and post-soaking losses and agrees with the previous work [12, 26] confirming that at present losses of WSC from soaking hay for a variety of times cannot be predicted according to hay species or WSC content. It is important therefore, to highlight to horse owners that WSC losses from soaking hay cannot be set nor predicted according to either soaking time or hay species. The best practical advice to owners who want to feed low WSC forage, is to purchase fibrous hay with a WSC content lower than 100g/kg DM or take a representative sample (from several different bales) and analyse the post soaked hay in order to achieve the optimum soaking time for reducing WSC in that hay.

There was also a highly variable response in bacteria growth (CFU/g) and in phyla, family and genera profiles across soaking times and hays. The similarity noted between the meadows hays in the dry samples continued post soaking with MC and MS producing more similar profiles after treatment compared with PRG. The quantity of bacteria growth across all 3 hays in this study was not as great as noted by Moore-Colyer and Fillery [15] who recorded a 5 fold increase in CFU/g when grass hay was soaked for 0.5 hours. The difference could be a factor of the degree of viability of the bacteria species on each of the hays or be due to the shorter wetting periods stimulating bacteria growth whereas longer soaking times can actually kill the bacteria (Daniels pers com). More work needs to be done to determine if there is an optimum soaking time to maximise WSC loss without increasing bacteria CFU/g. Little is currently known about the survival of feed bacteria through the equid gastro intestinal tract. The more hostile regions of the gut eg. the low pH in the pyloric region of the stomach may neutralise many feed bacteria but the fact that animals commonly suffer gastrointestinal upset post ingestion of contaminated foodstuffs suggest that bacteria load may have a major impact on gut health.

Bacteria growth was also not correlated with WSC content across the hays. Several studies have revealed [13,14, 41] that varying amounts of nutrients can be leached from leaves when the plant is exposed to water, but the plant features that may influence the degree of leaching are yet to be determined. It might be expected that leaching of WSC from plant material into a readily available solution would support bacteria growth, however, the correlation across all three hays showed that no relationship existed between the amounts of WSC leached from the hay and the total number of bacteria colony forming units on the hay.

Within the complex multifactorial relationship that exists between bacteria and the phyllosphere, there are bacteria that can increase the wettability of leaves by producing compounds with surfactant properties [42]. Fifty percent of the genus of metabolically diverse and wide niche colonizers Pseudomonas have been reported [43] to have this ability. One possible explanation for the degree of WSC leaching at the extended soaking times is an increase in the wettability which allows solubilisation and diffusion of substrates into the water. While no relationship was noted between wettability and the presence of Pseudomonas, it might be due to specific species of Pseudomonas present and /or other yet unidentified factors. However, this does not explain the differences noted between the 2 meadow hays and the PRG in the amount of WSC leached at 9 and 16 hours. The diversity of bacteria families was similar across the 3 hays, and the *Pseudomoadaceae* family were present in all 3 hays, yet the loss of WSC from the PRG for the 9 and 16 hours soaking was greater than the losses from the 2 meadow hays. Clearly other yet unidentified factors are influencing WSC loss and this requires further investigation in order to better understand the effect soaking has on WSC loss in hay.

### Soaked hay for horses

Although the disadvantages of soaking such as inconsistent WSC loss [12] mineral leaching [8,13] and increases in bacteria colony forming units [15] have been well documented, there is no information on the bacteria profile of common horse hays; how hay type influences bacteria profile, or how soaking might alter that profile. The fact that the Enterobacteriaceae family increased in MC9 compared with MCD, but was also high in PRGD, (contains the gram negative genera *Erwinia*, *E*. *coli*, *Shigella*, *Salmonella* and *Yersinia* all of which are potential pathogens), demonstrates the need for further investigation into bacteria profiles and abundancies in hay as to better understand the potential impact on horse digestive health.

Previous work has shown that soaked hay is less palatable [44,45] compared with haylage, dry, and steamed hay, although the reduction in palatability has not been hitherto associated with a reduced hygienic profile. Ericsson *et al*. [46] reported the gastric microbiome to be composed of a core of multiple small OTUs which may be readily influenced by external factors such as feed. Although the four major phyla (96% of the phyla present) noted in the three hays, *Proteobacteria*, *Cyanobacteria*, *Actinobacteria* and *Bacteriodetes* are present in the equid gut [46] they are in different proportions in the GIT than those noted in the hays in this study. This may offer a challenge to gastric physiology. One family, the *Enterobacteriaceae* comprised 25% of the proportion of bacteria families present in PRG and this family contains potential pathogens. Muller *et al*. [47] also recorded high levels (4.9 log ^10^ CFU/ g) of *Enterobacteria* in dry hay samples, thus clearly hay supports the growth of this family of bacteria. The longer soaking times of 9 hours in the MC hay caused an increase in this family, although the shorter soaking time of 1.5 hours decreased *Enterobacteriaceae* in the PRG hay. Clearly a larger number of hays need to be analysed to understand the shifting response to soaking in bacteria profiles. Soaking at different temperatures, influenced by time of year also needs to be investigated as this is likely to be influential in terms of individual bacteria species response.

## Conclusion

This is the first study to map the bacteria profile of horse hay and although the number of hays tested in this preliminary study was limited the results give an indication of a degree of commonality between hays, with similar phyla profiles of *Proteobacteria*, *Actinobacteria*, *Firmicutes and Cyanobacteria* comprising 96% of the bacteria present on the phyllosphere of all three hay types. The response to soaking was however, individual according to hay type, and no relationship existed between WSC leaching, the quantity of bacteria present post soaking, or the alterations in bacteria profiles. An increase was noted in the family of potential pathogens the *Enterobacteriaceae* in one of the Meadow hays but this was not mirrored in the other meadow hay or the perennial rye grass hay. The results from this preliminary trial shows that soaking hay to reduce WSC content is highly inconsistent, can increase bacteria numbers and may cause a rise in potential pathogenic bacteria in post-treated hay. Testing a wider variety of hays may elucidate a response pattern and provide some insight into the behaviour of nutrients and bacteria when hay is soaked, but at present the factors influencing nutrient leaching and microbial growth during soaking are still unclear and unpredictable.

## Supporting information

S1 Table Hay TVC WSC. (XLSX)

S2 Table Family Shannon, Jacard between hay and treatment comparisons. (XLSX)

S3 Table Full biome table raw data. (XLSX)

S4 Table ANOVA Phyla data. (XLSX)

S5 Table Raw Data Family Level DESeq2 paired t-test on time and hay comparisons. (XLSX)
